# Mobile Phone Text Messaging: Tool for Malaria Control in Africa

**DOI:** 10.1371/journal.pmed.1001176

**Published:** 2012-02-21

**Authors:** Dejan Zurovac, Ambrose O. Talisuna, Robert W. Snow

**Affiliations:** 1 Malaria Public Health and Epidemiology Group, Kenya Medical Research Institute-Wellcome Trust Research Program, Nairobi, Kenya; 2 Centre for Tropical Medicine, University of Oxford, John Radcliffe Hospital, Oxford, United Kingdom

## Abstract

Dejan Zurovac and colleagues discuss six areas where text messaging could improve the delivery of health services and health outcomes in malaria in Africa.

**Summary Points**

- Across many malaria-endemic areas in rural Africa, the communication gap between managers, health workers, and patients is a significant barrier to efficient malaria control.
- The rapid expansion of mobile network coverage and the widespread availability of basic handsets have the potential to substantively bridge the communication gap.
- Text messaging, as the least-expensive mobile phone function found on all handsets, could improve the delivery of health services and health outcomes.
- Six major areas of malaria control in which deficiencies are apparent and text messaging interventions could be beneficial are: (1) disease and treatment effectiveness surveillance, (2) monitoring of the availability of health commodities, (3) pharmacovigilance and post-marketing surveillance of the safety and quality of antimalarial drugs, (4) health worker adherence to guidelines, (5) patient adherence to medication regimens, and (6) post-treatment review.
- Text messages transmitting information from the periphery of the health systems to malaria control managers are in the first three malaria control areas: (1) disease and treatment effectiveness surveillance, (2) monitoring of the availability of health commodities, and (3) pharmacovigilance and post-marketing surveillance of the safety and quality of antimalarial medicines. Future projects in these three areas should demonstrate responses to data signals and comparative advantages with routine information systems.
- Text messages in the second three areas transmit information to health workers and patients to support the management of malaria patients by improving (4) health workers' adherence to guidelines, (5) patient adherence to medicines, and (6) post-treatment review. Future priorities in these areas are cost-effectiveness evaluations, qualitative research, and studies measuring impact on the processes of care and health outcomes.

## Malaria and Narrowing the Communication Gap in Africa

Across many malaria-endemic areas in rural Africa, health systems are weak, infrastructure is poor, and poverty is widespread. Traditionally, the communication gap between managers of health services, health workers at the periphery, and the patient population they serve has been a barrier to efficient service delivery [1]. This gap, however, has the potential to be bridged through the rapid expansion of mobile network coverage, availability of inexpensive handsets, and decreasing costs of mobile phone services [2],[3]. It has been estimated that over two-thirds of the population in Africa is covered by a mobile network with a penetration rate of 50%, reaching over half a billion mobile phone subscribers across the continent [3],[4]. The very nature of this coverage has resulted in various initiatives to alleviate poverty such as providing market information for rural farmers, assisting contract laborers to find employers [5], or using mobile phones as a virtual bank to pay for goods and services and to ensure immediate transfer of funds to remote areas [6]. The lack of immediate access to funds has been one of the economic barriers to accessing health services in rural areas [7]. Although they are not yet quantified, virtual bank initiatives and mobile money are likely to have a significant impact on access to travel funds to reach distal clinics.

Malaria has plagued Africa for centuries and exacted a heavy public health burden. International interest in its control has varied over the last 100 years. The last 10 years of the Roll Back Malaria Initiative, accompanied by substantial financial assistance from the Global Fund and bilateral agencies, have transformed the availability of preventative measures to poor communities in Africa, resulting in significant reductions in malaria across the continent [8],[9]. However, some of the greatest operational challenges to sustain this progress are in ensuring effective surveillance, continuous stocks of life-saving commodities, and adequate malaria case management [8],[10],[11].

**Figure 1:**
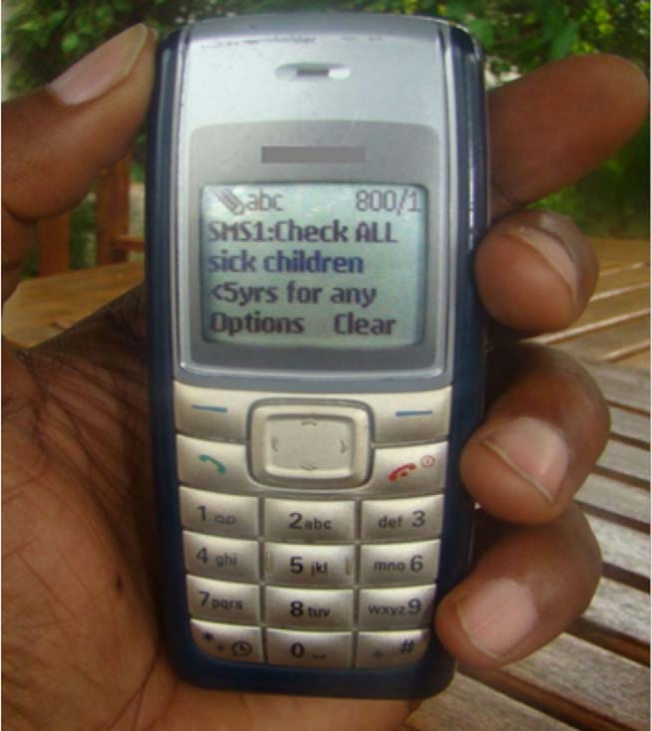
Example of mobile phones common in rural Africa.

The widespread use of SMS (short message service), the least-expensive mobile phone function, offers a solution that could rapidly overcome weaknesses in communication, potentially leading to improved delivery of health services and better health outcomes. Text messaging is particularly attractive because it is available on most basic handsets without the need for additional applications (Figure 1). SMS functions on a lower bandwidth than does voice, requires minimal skills in its use, offers automated delivery, and is personally convenient because of its asynchronous character [12],[13]. Despite the recognized potential of SMS technology, there are very few studies demonstrating the impact of text messaging in malaria control. For example, we searched Medline database and found only six studies reporting the use of text messaging within the malaria field in Africa (Table 1). The paucity of peer-reviewed studies limits our ability to compare and integrate possible effect sizes of new technologies on health systems or patient outcomes. As with any innovation, evidence from controlled trials is important to promote and effect policy change.

**Table 1:** Studies reporting use of text messaging for malaria control in Africa.

Country	Area of Malaria Control	No of HFs	Text Messaging Content	Dominant Text Message Flow	Reporting Frequency	Feasibility Shown	Potential Response	Reference
Zambia	Disease surveillance (foci detection)	13	Name of HF; Name of sender; No RDT tested; No RDT positive	Upstream	Weekly	Yes	Active screening; outbreak response	[20]
Madagascar	Disease surveillance (outbreak detection)	13	No of patient visits; No of patients meeting case-definition	Upstream	Daily	Yes	Outbreak response	[21]
Tanzania	Commodity monitoring	129	AL stock for each of 4 packs; stock quinine vials	Upstream	Weekly	Yes	Drug redistribution; Emergency orders	[22]
Uganda	Disease surveillance and commodity monitoring	147	26 malaria testing, treatment and ACT and RDT stock parameters	Upstream	Weekly	Yes	Drug redistribution; Emergency orders; Case-management corrections	[23]
Tanzania	Post-marketing surveillance	25	Patient demographic, date, type of event	Upstream	When occurred	Yes	Investigation of adverse drug reactions	[24]
Kenya	Health worker adherence	52	10 different case-management messages per week over 26 weeks	Downstream	Twice daily	Yes	Not applicable	[45]

ACT, artemisinin-based combination therapy; HF, health facility; RDT, rapid diagnostic test.

**Figure 2:**
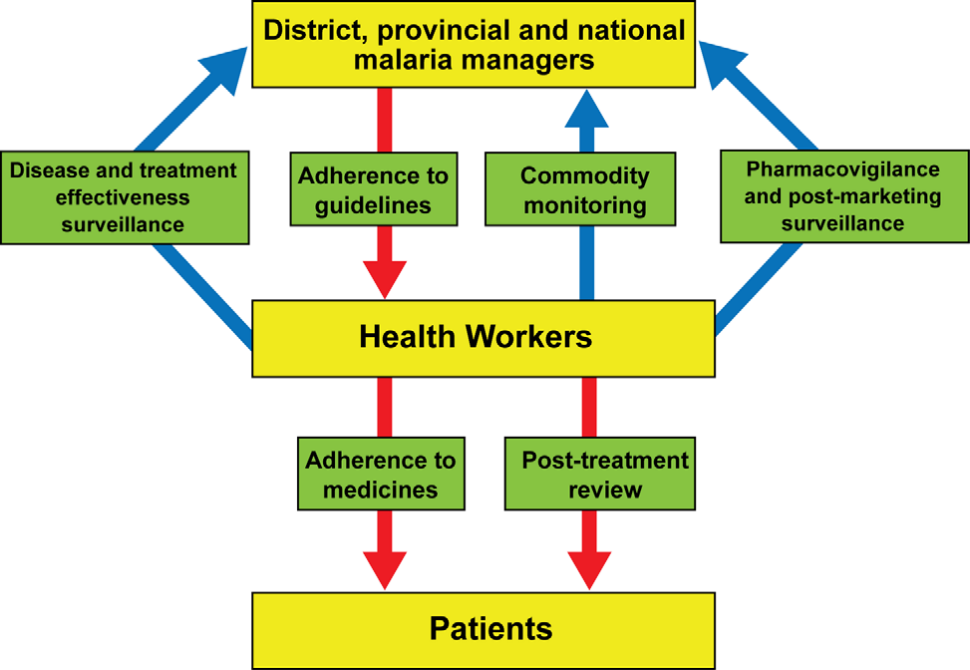
Potential applications of text messaging for routine malaria health service delivery. Blue arrows, dominant SMS communication targeting control managers; red arrows, dominant SMS communication targeting health workers and patients; green boxes, areas of intervention.

In this viewpoint we propose six major areas within malaria control in which solutions are urgently required and simple text messaging could offer the solution to improve routine delivery of health services (Figure 2). We consider text messaging interventions that have the potential for immediate scale-up, benefiting from the widespread availability of basic mobile phones that do not require installation and maintenance of additional applications (Figure 1). The next generations of mobile devices, such as “smartphones,” which are more complex, more expensive, and not yet widely available in rural Africa, are not considered here. We categorized interventions based on the dominant text messaging flow within the health system as those transmitting information from the periphery of the health systems to the control managers and those transmitting information to health workers and patients to support disease management (Figure 2).

## Text Messaging Interventions Transmitting Information to Malaria Control Managers

Effective malaria control depends on the range of routinely collected and timely reported health facility data. Three of these that can be broadly categorized as surveillance or monitoring activities are of particular importance following large scale implementations of malaria interventions across Africa [9]. They include (1) disease and treatment effectiveness surveillance, (2) monitoring the availability of commodities such as antimalarial medicines or rapid diagnostic tests, and (3) monitoring of adverse drug events (pharmacovigilance) and of the safety and quality of antimalarial products on the market (post-marketing surveillance). The latter is of particular importance given the threat of artemisinin tolerance recently detected in Southeast Asia and its imminent spread to the African continent; early detection tools are needed as part of treatment effectiveness surveillance [14].

Unfortunately, in many African countries, the routine health, logistics, and surveillance systems through which malaria data are reported in an “upstream” direction to managers are weak components of the health systems [15]–[18]. The validity and utility of information is compromised because the poor quality of source information, low reporting rates, delays in data acquisition, and lack of visibility of predefined unit signals and summary indicators preclude prompt responses to threats and emergencies such as malaria outbreaks, stock-outs of antimalarial drugs, presence of suboptimal drugs [19], or occurrences of severe adverse drug reactions and treatment failures.

The broad availability of mobile phones and with them the use of text messaging among health workers in rural areas could overcome delays, ensure nearly real-time data acquisition, and through computerized platforms make available pre-defined indicators to control managers. Such timely visibility could strengthen governance of scarce resources and should thereby result in prompt responses at different levels of the health system (in many cases, responses at the district level could be sufficient to mitigate the problem). For example, within our first proposed area of intervention—aiming to improve disease and treatment effectiveness surveillance—district supervisors could respond by initiating investigations of and targeted interventions to unusual upsurges of malaria, by reinforcing health workers' testing and treatment practices for detected discrepancies between test-positive and reported malaria cases, or by verifying reports of increased treatment failures and calling for urgent support for studies to confirm or rule out artemisinin resistance. Within our second area of intervention targeting availability of health commodities—district supervisors should be able to respond to threatening stock-outs of medicines or diagnostics by redistributing commodities between facilities or placing emergency orders. Finally, within the third intervention area—pharmacovigilance and post-marketing surveillance—suspected reports of adverse drug reactions and alerts of counterfeit drugs require prompt field verification, sample collection, and initiation of product investigations at the central level with the regular feedback provided to reporting clinicians.

In recognition of the possibilities of timely information transfer by text messaging, it is not surprising that nearly all pilot projects within the malaria field were indeed in the areas of surveillance and commodity monitoring (Table 1). Disease surveillance has been reported by SMS in Zambia [20] and Madagascar [21], and commodity monitoring in Tanzania [22]. In Uganda, the utility of SMS was assessed for both disease and commodities monitoring [23]. In Tanzania, the safety of antimalarial therapy was monitored [24]. The number of SMS reporting parameters ranged from four to 26. All text messages were sent from a health worker's personal mobile phone without additional software applications, and all projects demonstrated the feasibility of reporting real-time data, achieving high rates of reporting, and potential utility for control managers. However, with the exception of the Tanzanian projects, it was unclear how much health workers and the populations they serve benefited from these SMS-enhanced surveillance activities compared to those relying on routine information systems.

We envision that, in the near future, the role of SMS reporting will become most important and effective in areas where malaria public health emergencies are imminent, response mechanisms are clearly defined, and the impact of enhanced real-time reporting is proved superior to routine information systems. We emphasize responses at the health facility level, because failure to do so would both result in missed opportunities to save lives and risk health worker “reporting fatigue” regardless of whether reporting is based on the text messaging or on traditional methods.

## Text Messaging Interventions to Support Disease Management

High levels of patient adherence to antimalarial treatments and health worker adherence to malaria treatment guidelines are vital components of successful malaria case management. They help ensure good clinical outcomes for individual patients [25]–[27] and minimize the risk of drug resistance at the population level [28],[29]. Across Africa, clinical practices discordant with national malaria guidelines have been widely reported [30]–[33]. Patient nonadherence to lifesaving antimalarial therapies is also common [34]–[37].

Studies in developed countries [13],[38],[39] and an increasing number of rigorous trials in Africa [40],[41] have investigated the impact of text message reminders on patient adherence to long-term therapies for infectious and noncommunicable diseases, and all have shown encouraging results. To our knowledge, however, no study has yet been undertaken to investigate the use of SMS for widely used short-course therapies with complex dosing regimens in developing countries, such as those prescribed for malaria in Africa. Emphasis has traditionally been on more complex and expensive behavioral change initiatives to improve community- and clinic-based practices, including improved formulations and packaging of antimalarial drugs [42]–[44]. SMS reminders to caretakers and patients while the latter takes prescribed medications could improve compliance. This is one area of disease management where we see a need for carefully designed trials under various operational settings.

A further departure from traditional approaches to improving health worker performance has been recently tested in Kenya [45]. A randomized controlled trial at rural health facilities showed that SMS reminders sent to health workers' personal phones substantially improved their adherence to malaria guidelines, and that improvements were sustained after the end of the intervention. This is another area of intervention for which further work on optimizing intervention replication is necessary.

Post-treatment review of patients treated for malaria is a case-management component that deserves special attention. For malaria treatment reviews, day 3 outcomes are a valuable early warning signal when reported as part of the treatment effectiveness surveillance to detect emerging artemisinin resistance [14]. However, the feasibility of detecting this valuable information is possible only if patients do return to the facility for the post-treatment review. Unfortunately, across most of the outpatient settings in Africa, follow-up is one of the weakest components of the routine clinical process. In developed countries SMS messages have been widely used to remind patients of scheduled appointments [46],[47]. Similarly, more complex mobile phone applications have shown significant improvement in the follow-up of malaria patients in Thailand [48]. The same approaches should be tested in Africa as part of the SMS reminder package to improve patients' adherence to antimalarial treatment schedules.

The impact of text messaging applications in the areas of malaria adherence and post-treatment review is still unclear in Africa. Feasibility projects including rigorous cost-effectiveness evaluations and qualitative research to better understand determinants of the successes or failures of SMS interventions are urgently required. Importantly, even if text message reminders are shown to be simple, inexpensive, and effective in improving adherence they are unlikely to achieve perfect outcomes on their own. Therefore they should be seen as a booster to malaria care and not as a solution replacing basic programmatic inputs such as delivery of in-service training, guidelines and supportive supervision, clinical audits for health workers, or provision of child-friendly medicine formulations with pictorial inserts for patients.

## Barriers and Gaps to Adoption of Text Messaging in Routine Malaria Control in Africa

We have highlighted the scarcity of peer-reviewed studies and thus the absence of an evidence platform to gauge the effectiveness of text messaging for malaria control in Africa. SMS-based interventions should be held to the same evidence-based criteria as any other new tool for malaria control, even before considerations of policy adoption and implementation. Furthermore, there are several other adoption barriers that deserve attention. First, despite the focus of our interventions on the use of basic personal mobile phones and the least expensive SMS function, the actual implementation costs must be carefully determined as part of effectiveness studies. Second, high usability of mobile applications for health workers and patients in rural Africa is an important feasibility determinant. Other than the voice function, text messaging is the simplest and the most widely used technology function for which all of the reviewed studies have shown ease of use in reporting periodic data from the health system periphery to control managers. This however remains to be proved for interventions targeting individual patients, to whom a high facility workload or illiteracy may present a barrier. Third, although a substantial number of reporting parameters can be included in SMS interventions [23], these capacities are lower than in more complex software applications that can be installed on the new generations of mobile devices. To allow expansion of SMS-based interventions beyond malaria control, we call for reporting of a minimum number of critical surveillance parameters sufficient to trigger well-established emergency responses. Fourth, the proposed interventions do not depend on installation and maintenance of any software applications on the phone itself. There is, however, a technical requirement for development, hosting, and maintenance of computerized platforms that may not be locally available in all African settings. In addition, the reach of SMS-assisted innovation does depend on mobile network coverage. It is now possible using geographic information systems and cell tower locations to define marginalized health facilities and populations in malaria risk areas where SMS interventions are especially needed but are beyond the national coverage grid. This work must be undertaken in parallel to controlled trials to ensure an effective national policy platform. Finally, from the ownership perspective relevant for policy adoptions, the field of mobile applications in health, which was traditionally led by computer sciences, should evolve into multidisciplinary work including epidemiologists, social scientists, health systems researchers, and national policy makers from health, information technology, and development.

## Conclusions

We have suggested six areas of malaria control in which text messaging communication may improve delivery of services and health outcomes (Figure 1). For the three areas in which information is transmitted from the periphery of the health system to malaria control managers—disease and treatment effectiveness surveillance, monitoring the availability of health commodities, and pharmacovigilance and post-marketing surveillance of the safety and quality of antimalarial medicines—future priorities are projects demonstrating responses to detected data signals and comparative advantages with routine information systems. For the three areas in which transmitted information would help support management of malaria patients by improving health worker adherence to guidelines, patient adherence to medicines, and post-treatment review, future priorities are rigorous cost-effectiveness evaluations, qualitative research, and studies measuring impact on the processes of care and health outcomes.
